# Phaeohyphomycosis of the Eyelid: A Case Report

**DOI:** 10.7759/cureus.75186

**Published:** 2024-12-05

**Authors:** Suwarna Suman, Arushi Kumar

**Affiliations:** 1 Department of Ophthalmology, All India Institute of Medical Sciences, Jodhpur, Jodhpur, IND; 2 Department of Ear, Nose, Throat (ENT), Nalanda Medical College and Hospital, Patna, IND

**Keywords:** dematiaceous fungi, eyelid mass, opportunistic fungal infection, phaeohyphomycosis, subcutaneous phaeohyphomycosis

## Abstract

Phaeohyphomycosis is a fungal infection caused by dematiaceous fungi that presents as a superficial, cutaneous, subcutaneous, or systemic infection. Subcutaneous phaeohyphomycosis is the most common manifestation and presents as a subcutaneous nodule or cystic lesions and abscesses. It usually results from traumatic implantation of the saprophytic fungus from soil and vegetative matter; therefore, the commonest sites of infection are the extremities. Rarely, involvement of the eyelid is seen with extensive facial infection or as a part of multiple nodular lesions in the face, scalp, or other regions in cases of cutaneous/subcutaneous phaeohyphomycosis.

Here, we report a case of subcutaneous phaeohyphomycosis presenting as a solitary nodular lesion of the eyelid with extension into the anterior orbit in a 35-year-old immunocompetent male. The patient was successfully treated with combined surgical and medical therapy.

## Introduction

Ajello et al. in 1974 introduced the term phaeohyphomycosis to describe infections caused by hyphomycetous fungi (a group of conidial fungi characterized by the formation of asexual spores, the conidia, on conidiophores that are not contained in a fruiting body; commonly known as molds) that develop in the host tissues in the form of dark-walled dematiaceous septate mycelial elements [1]. Later, its definition was expanded by the author and included fungi Ascomycota as well [2]. Currently, phaeohyphomycosis refers to the infections caused by dematiaceous (pigmented fungi) containing melanin in their cell wall (phaeoid or darkly pigmented fungi), which develop as dark-walled, septate mycelia elements in their host's tissues [3]. Clinically, phaeohyphomycosis is classified as a superficial, cutaneous, subcutaneous, or systemic infection [4]. Superficial phaeohyphomycosis is confined to the stratum corneum with little or no tissue response or damage. For example, black piedra is caused by Piedraia hortae, characterized by hard, black, carbonaceous nodules that are firmly attached to the hair, and tinea nigra is caused by *Phaeoannellomyces werneckii*, involving the stratum corneum. Cutaneous and corneal phaeohyphomycosis involve keratinized tissues. Usually, non-living layers are involved with extensive tissue destruction [4].

Subcutaneous infection following trauma is one of the common forms of phaeohyphomycosis. In a literature review from China, subcutaneous infection was most commonly reported in 49% (85/174) of cases [5]. Subcutaneous phaeohyphomycosis usually presents as an asymptomatic solitary mass or nodule at the site of previous trauma like cuts, abrasions, or splinters [6]. Therefore, the commonest sites of infection are exposed body parts such as the feet, legs, hands, arms, and head [5-7]. The lesion may gradually enlarge to a granulomatous lesion with a central cavity (phaeohyphomycotic cyst) with intact overlying skin [8]. Mostly, the lesion remains localized, and multiple lesions are rare [6-8]. Disseminated invasive infections usually occur in patients with immunodeficiency [9]. Systemic phaeohyphomycosis includes central nervous system infection, pulmonary infection, sinusitis, eye disease, arthritis, osteomyelitis, fungemia, endocarditis, peritonitis, and gastrointestinal disease [9].

Several cases of subcutaneous phaeohyphomycosis involving the leg, foot, toes, arm, hand, wrist, waist, or buttock have been reported worldwide, more commonly in tropical and subtropical countries [4-6]. We report a case of subcutaneous phaeohyphomycosis presenting as a nodular lesion of the eyelid with extension into the anterior orbit in a 35-year-old immunocompetent male.

## Case presentation

**Figure 1:**
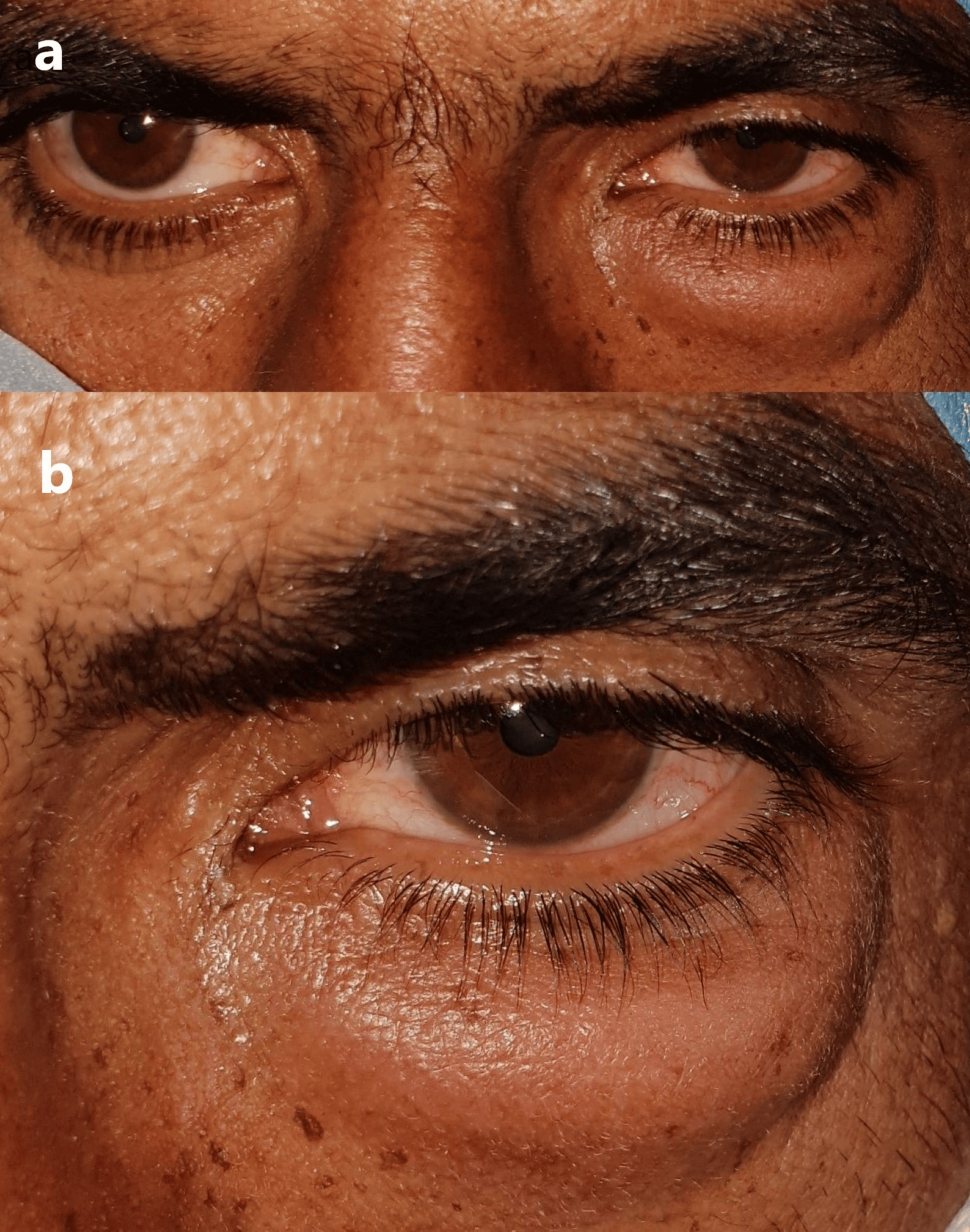
(a) A subcutaneous nodule of the left lower eyelid. (b) Close-up view

A 35-year-old male farmer presented with swelling of the lower eyelid in his left eye for two months. It was slowly and progressively increasing in size. There was no history of eye trauma or surgery, any foreign body or insect bite, or other lesions in the body. His medical and family history was not significant. He consulted a local doctor one week before, where he was prescribed the tablets amoxycillin (500 mg) + clavulanic acid (125 mg), one tab three times a day, and eye ointment moxifloxacin at night with no relief. Examination at presentation revealed a non-tender, firm, nodular swelling of the left lower eyelid around the inferior orbital margin. There was no deviation or displacement of the eye (Figure 1). The rest of the ocular examination in both eyes was within normal limits. The superficial lymph nodes were not enlarged. There was no evidence of any associated ocular or systemic condition predisposing to opportunistic infection.

**Figure 2:**
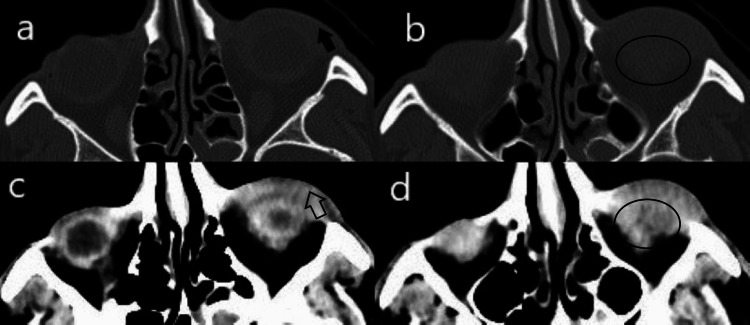
CT scan showing soft tissue mass lesion in left lower eyelid (arrow) with intraorbital extension (circle) in (a, b) thin bone and (c, d) thin plain axial section

Routine hematological and serological investigations were within normal limits. Non-contrast computed tomography scan revealed a well-defined soft tissue density (23 mm × 21 mm × 10 mm) involving the left lower preseptal space extending into adjacent extraconal space with surrounding fat stranding. No obvious bony erosion was seen (Figure 2).

**Figure 3:**
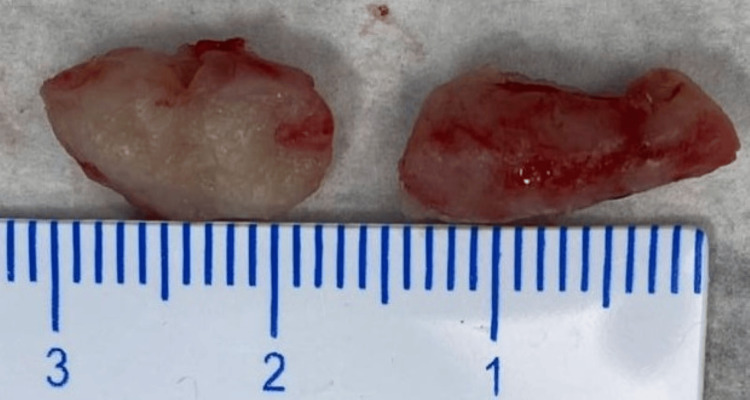
Excised soft tissue mass

An excisional biopsy was planned to confirm the diagnosis. The mass lesion was excised by anterior orbitotomy through an inferior lid crease incision. It was greyish-white in color, solid on the cut section, and firm in consistency (Figure 3). The postoperative period was uneventful.

The patient visited us three weeks after surgery with histopathology reports. Histopathological examination showed fibrocollagenous tissue bits with numerous non-necrotizing epithelioid cell granulomas and interspersed multinucleated giant cells. Within the giant cells, there are fragmented, slender fungal hyphae. Periodic acid Schiff and Gomori methenamine silver stains highlight these fungal profiles. There was an associated dense acute and chronic inflammatory infiltrate comprised of neutrophils, eosinophils, lymphocytes, and plasma cells. There was no evidence of malignancy. The findings were suggestive of fungal infection with granulomatous inflammation. At this stage, fungal culture and susceptibility testing were not technically possible because of the processing and fixing of the tissue with 10% buffered formaldehyde. The nuclear ribosomal internal transcribed spacer (ITS) sequencing could have been an option, as it can be performed from the fixated material, but unfortunately was not available at our institute.

On clinical examination, there was swelling around the scar mark. It was hard in consistency with ill defined margins. Tablet itraconazole 200 mg twice a day was started, and two weekly follow-ups were advised. After one month, there was no improvement in swelling. So, re-excision was performed along with medical treatment, and the specimen was sent for histopathological and microbiological examination. Histopathological examination showed few septate, brown-colored, irregularly branched fungal hyphae with plenty of hyphal swellings suggestive of phaeohyphomycosis. In KOH Mount, no fungal elements were seen. Fungal culture shows no fungal growth at 25°C and 37°C after four weeks of aerobic incubation. Therefore, the specific causative fungal strain of phaeohyphomycosis could not be identified.

**Figure 4:**
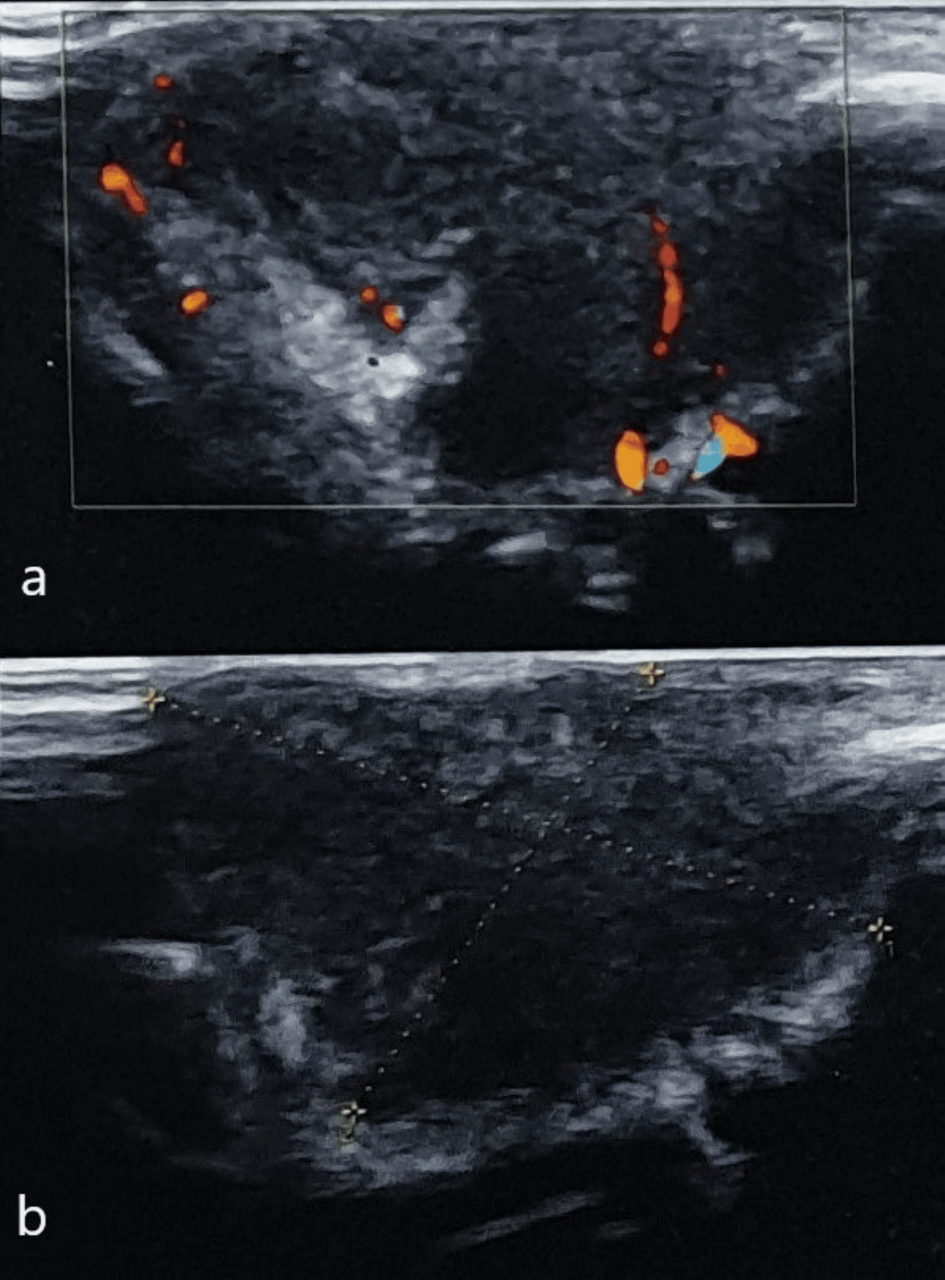
Ultrasound B scan showing (a) hypoechoic soft tissue mass lesion along the left lower orbital margin in the medial aspect and (b) regression of lesion after six weeks

Ultrasound orbit after two weeks postoperatively showed hyperechoic soft tissue density along the inferior orbital margin in the pre-septal region in the medial aspect. Antifungal treatment was continued, and an ultrasound orbit after six weeks showed some regression of the lesion (Figure 4). The patient was followed monthly in our outpatient department; blood counts and transaminase levels were checked every month. At the sixth month of itraconazole treatment, the lesion appeared to have completely regressed on clinical examination, and the patient was advised to have an ultrasound orbit to rule out any residual lesion. However, the patient was not willing, so he was advised to take itraconazole 100 mg twice a day for two months. On the 12-month follow-up, no recurrence was observed.

## Discussion

The dematiaceous fungi are a heterogeneous group of filamentous fungi having brown melanin or melanin-like pigments in the cell walls of their hyphae, conidia, or both [6]. These saprophytic plant pathogens are widely distributed in the environment and are found in soil, wood, and decomposing vegetative material, and they commonly infect humans through traumatic inoculation or as opportunistic infections in immunocompromised people [6,8]. About 148 species and 80 genera of dematiaceous fungi have been implicated in causing diseases in humans and animals [10]. The spectrum of diseases caused by the dematiaceous fungi includes chromoblastomycosis (infection of the skin and subcutaneous tissues), eumycotic mycetoma (characterized by draining sinuses that may involve deeper bones), and phaeohyphomycosis (a broad group of infections including superficial, cutaneous, subcutaneous, or systemic infections) [6,9].

Subcutaneous phaeohyphomycosis is commonly present as a solitary subcutaneous mass or nodule following trauma [6]. It may gradually increase in size to form a painless cystic abscess due to central necrosis of the nodule if not treated timely [6]. However, the overlying skin is usually spared [4,6]. The lesion usually remains localized, and lymphatic or hematogenous dissemination is rare [6,8]. Involvement of the eyelid is rarely seen in cases of extensive facial infection with multiple cutaneous and subcutaneous lesions in phaeohyphomycosis [11]. To the best of our knowledge, we could not find an isolated single subcutaneous nodular lesion of the eyelid with spared skin typically present in subcutaneous phaeohyphomycosis of extremities or other parts of the body. In this case, a solitary large subcutaneous nodule of the eyelid with a deeper extension into the orbit was seen. The overlying skin was spared. It was not preceded by a history of trauma, and there was no plant tissue present in the histopathological examination. However, there is a possibility of an unnoticed trivial injury as the patient is a farmer. He used to work in fields and live with domestic pets like bulls and chickens. There was no associated ocular or systemic condition predisposing to opportunistic infection.

In the eye, phaeohyphomycosis commonly causes keratitis, and the commonest mechanism of acquiring the infection is via implantation by organic matter [12]. The dematiaceous molds are reported as the third most common group of fungi, after Fusarium and Aspergillus species causing keratitis in India [6]. Curvularia species are the most common group of dematiaceous molds recovered from patients with mycotic keratitis in the United States [6,12]. Most commonly, infection was acquired by the traumatic implantation of spores into the corneal epithelium [6,12]. In a review study from China, Curvularia species were the second most common species after Colletotrichum species [5]. Rarely, cases of endophthalmitis, subretinal abscess, conjunctival, subconjunctival, orbital, and periocular lesions are reported, and both endogenous and exogenous infections have been implicated in ocular Phaeohyphomycosis [13-15].

Phaeohyphomycosis is an opportunistic infection. The melanin pigment common to all dematiaceous fungi has been implicated as a virulence factor that acts by inhibiting phagocytosis, even in immunocompetent hosts. Melanin acts as a scavenger of free radicals and hypochlorite and inhibits hydrolytic enzymes produced by phagocytic cells that normally kill most organisms [16,17].

The diagnosis of phaeohyphomycosis is based on the clinical presentation, histopathological demonstration of tissue invasion by fungi, and fungal isolation from culture. The fungal hyphae in the host tissue seem brown in unstained preparations. The hematoxylin and eosin staining and Gomori methenamine silver staining are sensitive to the detection of fungal cells when fungal elements are scarce. The Masson-Fontana stain is useful for detecting the melanin of dematiaceous fungi. It stains the fungal elements brownish-black [5]. Phaeohyphomycosis can be differentiated from other types of dematiaceous fungi on the basis of morphological characteristics of hyphae, such as the presence of grain in an eumycetoma or thick-walled muriform cells (sclerotic bodies) in tissues, which are characteristics of chromoblastomycosis [6]. However, the species of dematiaceous fungi cannot be identified only by histopathological examination; fungal isolation on culture medium is essential. Sabouraud dextrose agar is commonly used for fungal isolation; however, several clinically important species showed poor spore formation. Plant-based media such as potato dextrose agar, corn, and oat dextrose agar may be used to stimulate sporulation in dematiaceous fungi. If the fungal species cannot be identified by the culture isolation and microscopic examination, which depends on the recognition of typical reproductive structures, such as conidia, and their relationship to the fungal hyphae, molecular techniques are useful to identify fungi by comparing DNA sequences from an unidentified fungus with those from well-pedigreed isolates. Sequence analysis of the ITS and D1/D2 regions of rDNA is recommended for effective and accurate molecular identification of dematiaceous fungi. ITS has been reported as the standard fungal barcode [5]. In this case, fungal culture showed no fungal growth at 25°C and 37°C after four weeks of aerobic incubation. This may be because of continuous antifungal therapy for the past month before re-excision was done.

Differential diagnoses with similar clinical features are malignant nodular lesions of the eyelid, tuberculosis, pseudotumor, lymphoma, neurofibroma of the eyelid and orbit, etc. A negative Mantoux test, normal hematological investigations, no lymphadenopathy and similar lesions in the body, and imaging findings helped to differentiate from these conditions. However, a solitary eyelid lesion, although a rare presentation, is difficult to diagnose and essentially needs histopathology and microbiological examination for confirmatory diagnosis.

The treatment of subcutaneous phaeohyphomycosis is not standardized. Treatment options include surgical excision with or without antifungal treatment. A complete surgical excision of the lesion is usually successful, and antifungal treatment is not required. Incomplete removal of involved tissues or incision and drainage procedures may result in recurrence [18]. Antifungal agents can be added in patients with multiple lesions in different sites, indicating hematogenic dissemination or complete surgical removal of the lesion is not possible. An azole antifungal agent is frequently used in combination with surgery [17]. Itraconazole has been the preferred choice for antifungal systemic therapy, as the in vitro susceptibility of most strains of dematiaceous fungi to this drug is high [19]. There are only a few case reports of primary subcutaneous phaeohyphomycosis in immunocompetent patients in the literature to study the treatment outcome. A successful outcome is documented with electrocoagulation, surgical excision, antifungal therapy, and frequently with a combination of surgical excision and antifungal agents [20]. Although, in the immunocompetent individual, localized nodular or cystic form of subcutaneous pheohyphomycosis can be treated by complete surgical excision, however, in this patient, there was recurrence within three weeks of surgery, which may be because of some residual lesion or there was invasion of mycelial elements in the surrounding orbital tissue seen as fat stranding in the CT scan. Initially, no improvement was seen in the recurrent lesion with oral antifungal treatment; however, a successful response was received with re-excision and an eight-month itraconazole treatment. Similarly, a case of eyelid subconjunctival infections from *Exophiala phaeomuriformis* with a history of Stevens-Johnson syndrome is reported in which the progression of lesions was noted after two months of oral itraconazole (400 mg daily). After re-excision and continued itraconazole (200 mg daily), an apparent resolution was noted in four months. However, due to the patient’s self-termination of antifungal therapy, recurrence occurred after five months. The patient finally achieved complete resolution of the infection after a third excisional biopsy of all lesions and oral terbinafine 250 mg BID and posaconazole 300 mg daily treatment for three months [15].

Progression and relapses are troublesome in immunosuppressed patients. Sharkey et al. reported successful outcomes (stabilization of progressive infection, improvement, and remission) in 11 out of 17 patients with phaeohyphomycosis; 15 patients had previously failed responses to other antifungal agents (amphotericin B, fluconazole, ketoconazole, and miconazole). Six patients have had unsuccessful outcomes (progression in four, relapse in one patient, culture positive but clinically stable in one patient) attributed to severe underlying immunosuppression, concurrent uncontrolled bacterial infection, and insufficient duration of treatment. Out of 11 successes, four were immunosuppressed patients and seven were immunocompetent, and out of six patients failing itraconazole treatment, four had some form of systemic immune suppression [21]. Treatment of persistent infection and relapses in patients with immunodeficiency is frustrating both for the patient and the clinician. Local excision with post-surgical oral itraconazole has been reported to be successful in lung transplant recipients with recurrent subcutaneous phaeohyphomycosis [22].

Amphotericin B, fluconazole, ketoconazole, and 5-fluorocytosine have been considered as alternative therapies for patients with severe disease, poor response, or hepatotoxicity to itraconazole [17]. The new triazole agents, namely voriconazole and posaconazole, are also effective against many dematiaceous molds in vitro [19,23].

This case describes a rare presentation of subcutaneous phaeohyphomycosis of the eyelid with multiple recurrences in an immunocompetent adult male. Although we could not identify the specific causative fungal strain of phaeohyphomycosis. A microbiological examination with biopsy should be advised, as a soft tissue mass lesion of the eyelid or orbit may be of infective origin. Progression and relapses may be difficult to treat even in immunocompetent adults, as in this case. However, a successful outcome was achieved with a long course of antifungal treatment after surgical excision in this patient.

## Conclusions

This case reports an unusual presentation of phaeohyphomycosis involving the eyelid with extension into the anterior orbit in an immunocompetent individual. Microbiological examination with biopsy is recommended in cases of soft tissue mass lesions of the eyelid and orbit even if there is no history of trauma or systemic diseases predisposing to opportunistic infections. In case of a negative fungal culture, molecular techniques are useful to identify the fungal species; however, availability is an issue. Surgical excision alone or in combination with antifungal agents is usually successful; however, the relapses after discontinuation are a concern. A long course of antifungal treatment after surgical excision may be needed to achieve a successful outcome in patients with recurrences. The optimal dose and duration of antifungal agents need to be standardized.
